# Inner hair cell stereocilia are embedded in the tectorial membrane

**DOI:** 10.1038/s41467-021-22870-1

**Published:** 2021-05-10

**Authors:** Pierre Hakizimana, Anders Fridberger

**Affiliations:** Department of Biomedical and Clinical Sciences (BKV), Linköping University, Linköping, Sweden

**Keywords:** Calcium signalling, Cochlea, Hair cell

## Abstract

Mammalian hearing depends on sound-evoked displacements of the stereocilia of inner hair cells (IHCs), which cause the endogenous mechanoelectrical transducer channels to conduct inward currents of cations including Ca^2+^. Due to their presumed lack of contacts with the overlaying tectorial membrane (TM), the putative stimulation mechanism for these stereocilia is by means of the viscous drag of the surrounding endolymph. However, despite numerous efforts to characterize the TM by electron microscopy and other techniques, the exact IHC stereocilia-TM relationship remains elusive. Here we show that Ca^2+^-rich filamentous structures, that we call Ca^2+^ ducts, connect the TM to the IHC stereocilia to enable mechanical stimulation by the TM while also ensuring the stereocilia access to TM Ca^2+^. Our results call for a reassessment of the stimulation mechanism for the IHC stereocilia and the TM role in hearing.

## Abstract (web summary)

Hearing requires inner hair cell (IHC) stereocilia deflection, believed to result from hydrodynamic coupling due to the lack of contact with the tectorial membrane (TM). Here the authors show that IHC stereocilia are TM-embedded, and calcium rich structures in TM may facilitate sound transduction.

## Introduction

Sound transduction into nerve signals happens when sound-evoked vibrations of the stereocilia of the inner hair cells (IHCs) and outer hair cells (OHCs) cause an indiscriminate entry of K^+^ and Ca^2+^ into the cells through open mechanoelectrical transducer (MET) channels^1–5^. These currents activate the OHC-based cochlear amplifier, essential for hearing faints sounds^6,7^ and cause the IHCs to produce electrical discharges in the auditory nerve^8,9^.

Patch-clamp recordings from isolated hair cells have established that extracellular [Ca^2+^], which a recent discovery of high [Ca^2+^] in the tectorial membrane (TM)^10^ helped estimate at 40–150 µM^11^, contributes to the regulation of hearing sensitivity.

Because the stereocilia of OHCs are attached to the TM^12^, their motions are proportional to the basilar membrane (BM) displacements^13,14^.

However, the IHC stereocilia stimulation mechanism is poorly understood.

Comparison of cochlear microphonic (CM) recordings in OHC-less and control guinea pigs in the early 70s suggested that the IHC stereocilia must be freestanding from the TM because their responses depended on the BM velocity^15,16^.

This generally agrees with the intracellular recordings from the IHCs that showed phase difference relative to CM recordings^17^. However, the obvious constraints imposed by the insertion of the recording electrodes^18^ might complicate the interpretation of these data.

While electron micrographs of the organ of Corti (OC) from the guinea pig freeze-dried samples have showed a good tissue preservation, the TM was detached from both the OHC and IHC stereocilia^19^. Today, the preservation of the TM-stereocilia relationship in fixed tissues remains a challenge^20^.

Here we use reflected light and fluorescence confocal microscopy to investigate the TM-stereocilia relationship in the guinea pig. We show that IHC and OHC stereocilia are similarly TM-embedded and that Ca^2+^ ducts connect the TM to the stereocilia of IHCs and OHCs.

Our results challenge the classic anatomical representation of the OC^21^ and show that the TM-embedment enables the stereocilia of OHCs and IHCs to directly access the TM mechanical stimulation and Ca^2+^.

## Results

### IHC stereocilia are TM-embedded

**Fig. 1:**
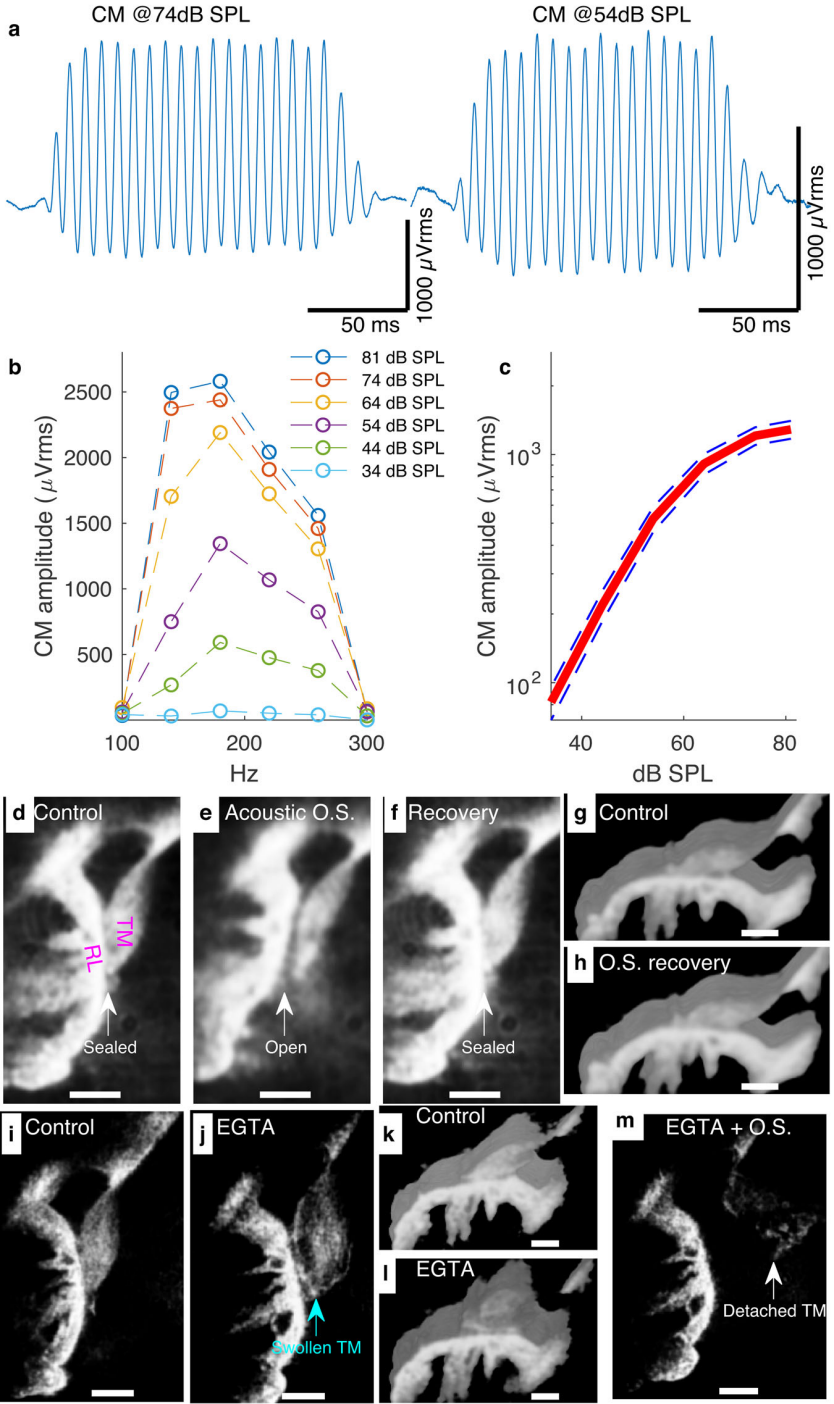
The TM interacts tightly with the RL including in the IHC region. **a**–**c** Effect of sound pressure level (SPL) on CM amplitude in the cochlear apical region in the temporal bone preparation from the guinea pig. When the eardrum is acoustically stimulated by a speaker inserted in the ear canal, the hair cells produce the CM (the individual examples shown in **a** were recorded in response to a 180-Hz tone burst at 74 and 54 dB SPL respectively). The peak-to-peak amplitudes from the raw CM waveforms were plotted against frequency and for several dB SPL values to generate the frequency-tuning curves shown in **b** (the individual curves shown in **b** are from the same preparation as **a**). The CM peak-to-peak amplitudes associated with the frequency-tuning curves were then averaged for 26 preparations and plotted against dB SPL to produce the plot shown in **c** (solid line, mean; error bar, s.e.m). Source data are provided as a Source data file. **d** Reflected light confocal image of the OC. Note the sealed TM-RL interface including within the IHC region. **e** Acoustic overstimulation (OS)-induced contraction of the OC exposed a TM-RL gap. **f** When the OS stopped, the TM-RL gap resealed. **g** 3D reconstruction (29 z-stacks with a spacing of 1 µm) of the OC before acoustic overstimulation. The image was rotated to show the opposite side of the image shown in **a**. **h** 3D reconstruction (26 z-stacks with a spacing of 1 µm) of the OC after the preparation was allowed to recover from OS. **i** Image of the OC showing a tight TM-RL interface before EGTA treatment. **j** Upon EGTA injection, the TM swelled markedly, which exposed a TM-RL gap. **k**, **l** 3D reconstructions of the OC before (**k**) and after EGTA injection (**l**) show that the TM swelling associated with Ca^2+^ removal by EGTA caused the TM to loosen from the RL (both reconstructions were obtained from 41 z-stacks with a spacing of 1 µm). **m** When the EGTA injection was followed by OS, the TM detached from the RL. All scalebars 50 µm. Data in **d**–**h** were representative of 37 different preparations. Data in **i**–**l** were representative of six preparations; Data in **m** are representative of five preparations.

Pioneered in the 80s^22^, the temporal bone preparation from the guinea pig makes it possible to investigate sound transduction in functional and largely intact OC^10,23–26^. Its CM recordings exhibit amplitudes and nonlinearity (Fig. 1a, b, c) generally consistent with those measured in living animals^27^. In addition, its cochlear mechanical responses are consistent with those obtained in vivo in intact cochlea^28^ and in vivo OC confocal imaging^29^ has confirmed that the hair cell morphology is well preserved in our preparation. This suggests that the native morphological organization and physiology of the OC and the TM, are most likely preserved in this preparation, making it suitable for investigating TM-IHC stereocilia relationships.

Although the TM appears nearly completely transparent to white light^30^, we realized recently that it reflects the green laser light, which we used to acquire confocal images of the TM and OC in situ. Staining with fluorescence dyes is not necessary and therefore image acquisition can be achieved through an intact Reissner’s membrane.

In the reflected light images, the reticular lamina (RL) was brighter than the TM, making it easier to distinguish an interface line between the two structures, with no obvious gap between them (Fig. 1d). This is consistent with the fact that the stereocilia in the OHC region are attached to the TM^12,31^. However, the TM-RL attachment, which contrasts with the traditional schematic representation of the OC^21^, indicates in addition that the OHC stereocilia are TM-embedded. Furthermore, the TM-RL attachment in the IHC region was surprising and suggests that the IHC stereocilia, too, are TM-embedded.

To test the TM-RL attachment strength, we stimulated the OC with a sound intensity of 103 dB sound pressure level (SPL), which causes the RL to move towards the BM^32^ (see Supplementary Movie 1). Surprisingly, the TM was pulled along while its shared interface with the RL, including in the IHC region, remained tight, causing the TM to drastically compress as it adjusted to the deformation of the OC (Supplementary Movie 1). This behavior was seen in all 37 preparations tested and indicated that the TM and RL were firmly attached in the IHC and OHC regions alike.

Immediately after the acoustic overstimulation stopped, the TM relaxed faster than the RL, causing a TM-RL gap to briefly appear (Supplementary Movie 1 and Fig. 1e), suggesting that the TM-RL attachments were overstretched by the OC contraction. However, this gap recovered (Supplementary Movie 1, compare Fig. 1d–f and Fig. 1g vs. h), indicating that it represented rather a pathological state.

Ca^2+^ is required for the integrity of various sensory structures of the OC^33,34^ but its effect on the TM integrity is poorly documented. Swapping endolymph with perilymph affected TM thickness but the results largely varied from one study to another^35,36^. Millimolar concentrations of Ca^2+^-chelating agents are routinely used to remove the TM from the RL^37^, but micromolar concentrations deplete TM Ca^2+^ without detaching the TM and had a reversible effect on mechanotransduction^10^. This suggests that low levels of Ca^2+^-chelating agents could be used to investigate Ca^2+^ depletion effect on the TM relationships with the RL and stereocilia in functional OC.

Figure 1i and Supplementary Movie 2 show that before ethylene glycol-bis(2-aminoethylether)-*N*,*N*,*N*′,*N*′-tetraacetic acid (EGTA) application, the TM rested tightly on the RL. However, upon 100 µM EGTA application through a thin glass electrode, the TM swelled rapidly, causing its normally “biconvex” shape to look more circular (Fig. 1j, Supplementary Movie 2); a behavior consistent in six preparations tested. These shape changes also caused a small TM-RL gap to appear in the OHC region (Fig. 1j, Fig. 1k vs. 1l, and Supplementary Movie 2). Although these changes were largely reversible (Supplementary Movie 2), they also suggested that Ca^2+^ depletion caused the TM interactions with the RL and stereocilia to weaken.

To investigate this possibility, we subjected the TM-RL junction to acoustic overstimulation in five of the preparations above immediately after EGTA application.

As shown by Supplementary Movie 2, after a brief acoustic overstimulation, the TM detached from the RL in all five preparations tested. Typically, when overstimulation stopped, the TM-RL attachments tended to recover (Supplementary Movie 2) but repeated EGTA injections combined with overstimulation eventually caused the TM to permanently detach from the RL, leaving the OC TM-less (Fig. 1m and Supplementary Movie 2). This indicates that the TM-RL attachments were robust but also Ca^2+^-dependent.

To gain a deeper insight into the TM-stereocilia relationship, we combined reflected light and fluorescence confocal imaging after OC staining with the membrane dye di-3-aneppdhq^10^.

**Fig. 2:**
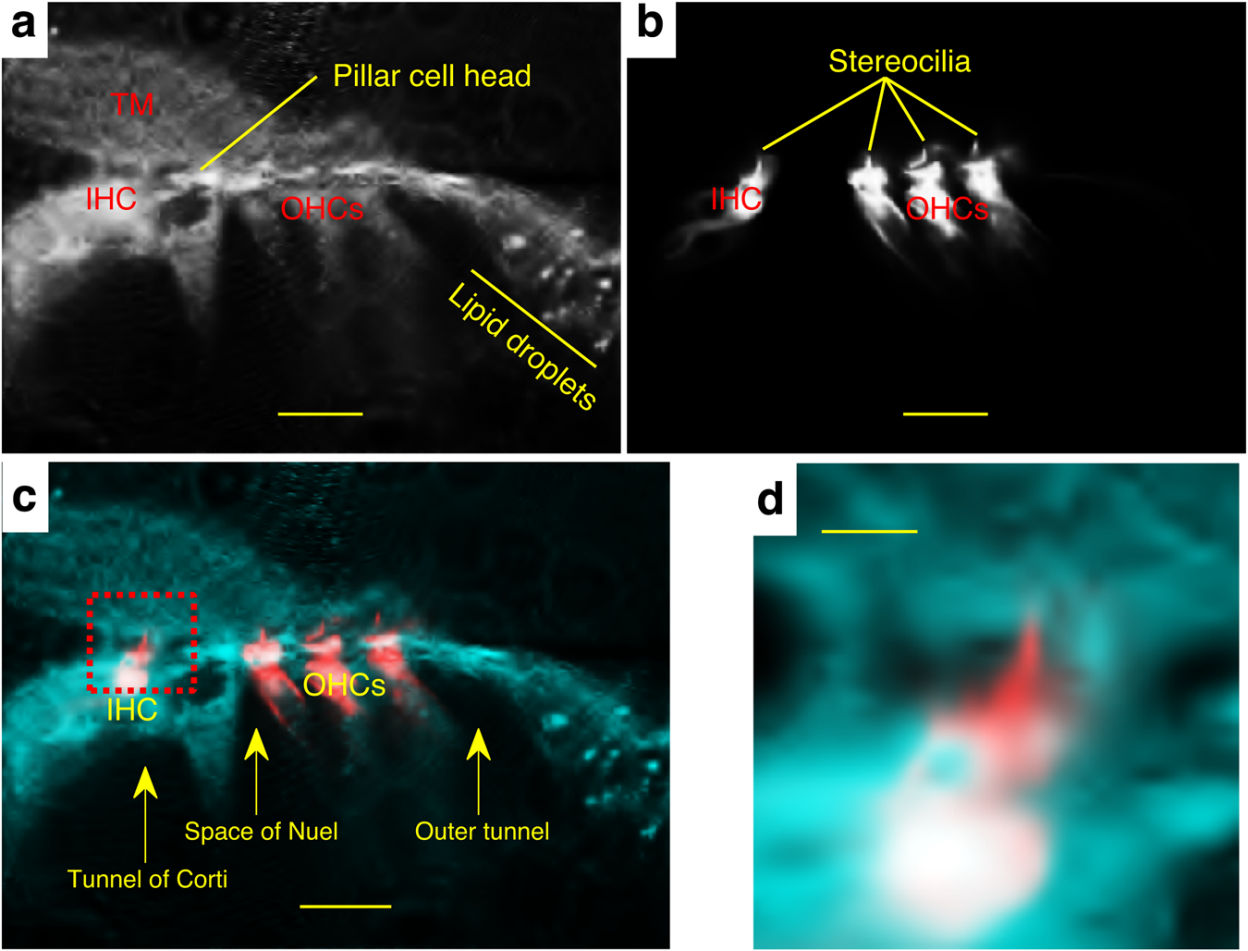
IHC and OHC stereocilia bundles alike are TM-embedded. **a** Reflected light confocal image of the OC showing tight interaction between the TM and RL. **b** Fluorescence confocal image of the same focal plane as in **a** after staining the OC with the membrane dye di-3-aneppdhq. **c** Overlay of images from **a** and **b** revealed that stereocilia of IHCs are TM-embedded in a similar fashion as the OHC stereocilia. **d** A closeup view on the IHC region (see red square in **c**) highlights the IHC stereocilia embedment within the TM. Scalebars: 25 µm in **a**, **b**, and **c**; 5 µm in **d**. Data in **a**–**d** were representative of ten different preparations.

High-resolution reflected light confocal images showed that the TM rested on the OC, where it intimately interacted with the RL, seen as a bright structure that spans from the inner phalangeal cells to the Hensen’s cells (Fig. 2a). A close inspection of the RL revealed weaker reflectivity zones in the OHC regions that delimited adjacent cuticular plates of the three OHC rows (Fig. 2a), in direct contact with the TM.

Surprisingly, the head region of the pillar cells was poorly reflective (Fig. 2a). A survey of the literature showed that this region is highly enriched in actin filaments^38^, a characteristic shared with the stereocilia^38^. This similarity could explain why both structure types had a poor reflectivity (Fig. 2a).

In the fluorescence channel, di-3-aneppdhq-stained the hair cells and their stereocilia^10^ (Fig. 2b).

Overlay of the reflected light and fluorescence channels showed that the OHC somatic and cuticular plate signals from both channels matched (Fig. 2c, d). In addition, the overlay demonstrated that the IHC stereocilia were TM-embedded (Fig. 2d). The image also shows that the OHC stereocilia were not merely attached to the TM but were rather TM-embedded (Fig. 2c).

### Ca^2+^ ducts ensure stereocilia access to TM Ca^2+^

To further investigate the TM-stereocilia relationships, we used a Ca^2+^ ratiometric indicator where Cal-520L, a bright and sensitive low affinity Ca^2+^ dye, and Cy5, a bright Ca^2+^-insensitive fluorophore, were conjugated to a 10 kDa-dextran. Because Cal-520L/Cy5 were spectrally independent, their presence made the conjugate a reliable ratiometric Ca^2+^ indicator. Typically, 2D OC confocal images associated with the two fluorophores were acquired simultaneously within minutes of the indicator injection.

**Fig. 3:**
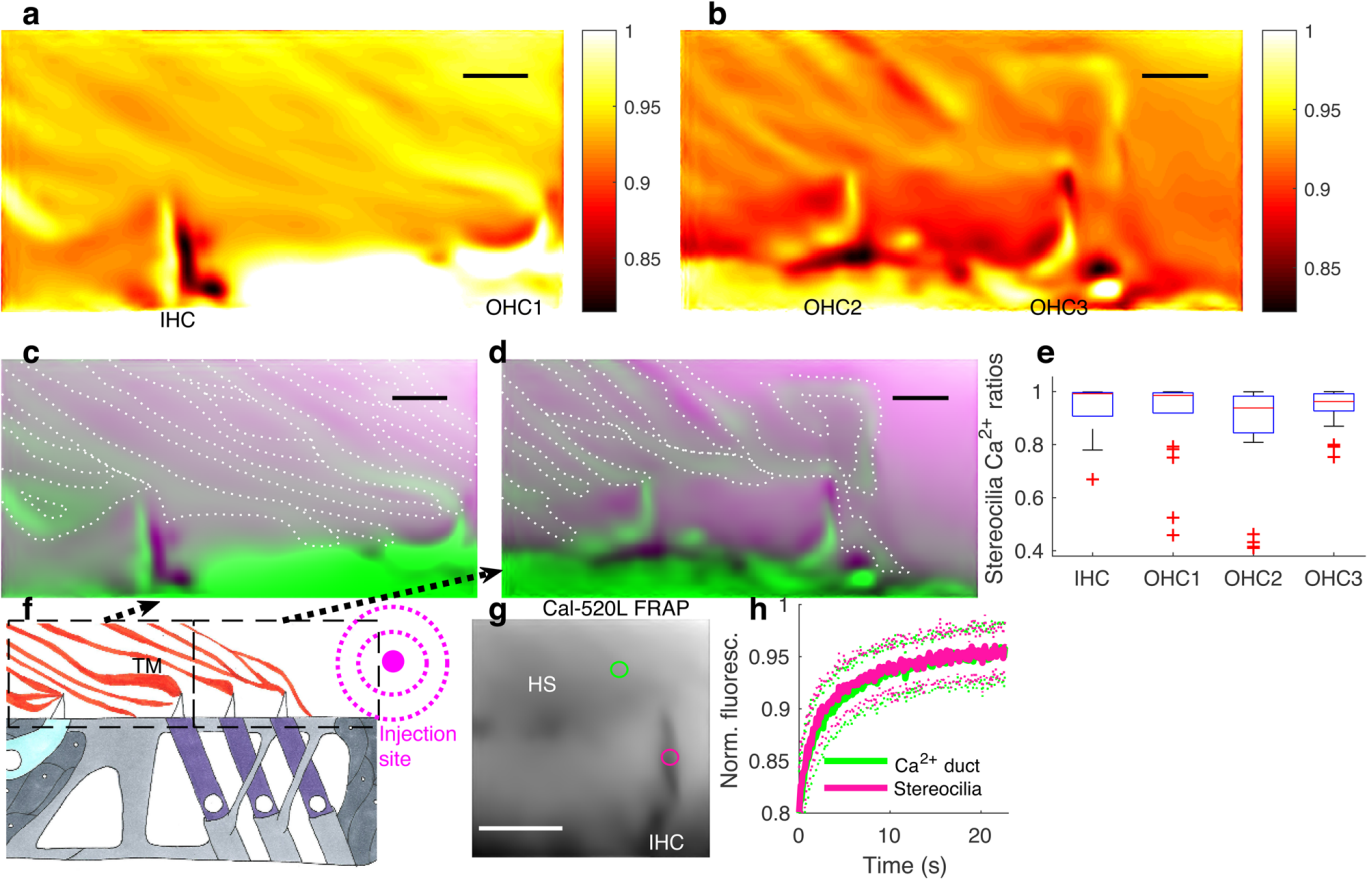
Ca^2+^ ducts connect the TM to the IHC and OHC stereocilia. **a**, **b** Ca^2+^ ratios, revealed Ca^2+^-rich ducts that connect the TM to the stereocilia of IHCs and row 1 OHCs (**a**); row 2 and 3 OHCs (**b**), respectively. **c** Overlay of the Ca^2+^ ratio image (green) from **a** with the corresponding reference image (Cy5 signal, violet) to highlight the contacts between the stereocilia of the IHCs and row 1 OHCs with the Ca^2+^ ducts. Dotted white lines were manually drawn to depict the boundaries of the Ca^2+^ ducts. **d** Same as in **c**, for row 2 and row 3 OHC regions. **e** Ca^2+^ ratios in the stereocilia bundles of different hair cell types. OHC1, row 1 OHCs; OHC2, row 2 OHCs; OHC3, row 3 OHCs. For IHCs, *n* = 19 IHCs from 11 preparations; for OHC1, *n* = 22 row 1 OHCs from ten preparations; for OHC2, *n* = 34 row 2 OHCs from eight preparations; for OHC3, *n* = 33 row 3 OHCs from eight preparations. Box-and-whiskers plot represents the maxima, 75th percentile, median, 25th percentile, and minima. Source data are provided as a Source data file. **f** OC schematic highlighting the regions confocally imaged to obtain images shown in **a**–**d** and injection site. **g**, **h** Single channel Cal-520L confocal image of the IHC stereocilia and the associated Ca^2+^ duct-rich TM region. The green and magenta ROIs (**g**) were used to investigate Ca^2+^ mobility in the Ca^2+^ duct-rich region and the IHC stereocilia, respectively by fluorescence recovery after photobleaching (FRAP). Averaged FRAP data associated with the ROIs shown in **h** (solid lines, means; error bars, s.d.). Source data are provided as a Source data file. For data in **g**, **h**, *n* = 41 same-location TM-IHC stereocilia from five different preparations. Scalebars 5 µm.

To visualize purely Ca^2+^-dependent signals, the Cal-520L and Cy5 2D confocal images were divided on a pixel-by-pixel basis, which resulted in ratiometric images of the OC, where the ratio in each pixel depends on Ca^2+^ (Fig. 3a, b).

As shown by Fig. 3a–d, Ca^2+^ ratiometric imaging produced Ca^2+^ signals within the stereocilia while also revealing high Ca^2+^ filamentous structures, or Ca^2+^ ducts, that radially spanned the TM to attach to the IHC and OHC stereocilia.

Although most of these Ca^2+^ ducts ran parallel to each other, they also branched occasionally, especially closer to the stereocilia attachment points, where they contacted stereocilia bundles in IHCs, row 1 OHCs, row 2 OHCs, and row 3 OHCs (Fig. 3a–d) and these findings were consistent in several locations of the cochlear apical region imaged in 12 preparations.

Since the Ca^2+^ concentration in the stereocilia has remained elusive despite its critical importance for hearing^11^, we used the ratiometric images to quantify the stereocilia bundle Ca^2+^ratios (Fig. 3e). In IHCs, the Ca^2+^ ratio was 0.94 ± 0.02 (*n* = 19 IHCs from 11 preparations, mean ± s.e.m.). In row 1 OHCs, the Ca^2+^ ratio was 0.91 ± 0.03 (mean ± s.e.m.; *n* = 22 row 1 OHCs from ten preparations). In row 2 OHCs, the Ca^2+^ ratio was 0.88 ± 0.03 (mean ± s.e.m.; *n* = 34 row 2 OHCs from eight preparations). In row 3 OHCs, the Ca^2+^ ratio was 0.94 ± 0.01 (mean ± s.e.m.; *n* = 33 row 3 OHCs from eight preparations). The Ca^2+^ ratio differences between the stereocilia types were not statistically significant (*p* = 0.1, Kruskal–Wallis test).

Interestingly, the Ca^2+^ indicator produced a signal in the cell bodies (Fig. 3a–d), possibly due to the accumulation of the indicator from the injection site (Fig. 3f) via endocytosis typical to dextrans^39^.

To determine whether Ca^2+^ from the ducts is transported to the stereocilia, we assessed Ca^2+^ mobility in the ducts relative to the stereocilia. Specifically, we monitored the fluorescence recovery of the dextran-conjugated Ca^2+^ dye Cal-520L after photobleaching (FRAP, reviewed in^40^) a 1 micron-wide spot within a Ca^2+^ duct-rich region and in same-location IHC stereocilia near the center of the bundle (Fig. 3g).

After photobleaching the Ca^2+^ signal (i.e., Cal-520L fluorescence), it recovered rapidly in both regions of interest (Fig. 3h). On average, the mobile fractions were 0.75 ± 0.01 and 0.76 ± 0.02 in the stereocilia and same-location Ca^2+^ duct region, respectively (mean ± s.e.m, *n* = 41 same-location TM-IHC stereocilia from five preparations). The difference was not statistically significant (*p* = 0.7, Wilcoxon signed-rank test). The recovery halftime in Ca^2+^ duct region was 2.89 ± 0.11 s (mean ± s.e.m, *n* = 41 same-location TM-IHC stereocilia from five preparations). For the stereocilia bundles, the recovery halftime was significantly shorter, i.e., 2.58 ± 0.10 s (*p* = 0.002, Wilcoxon signed-rank test).

The high mobile fraction of as much as 75–76% suggests that much of the Ca^2+^ in the stereocilia and Ca^2+^ ducts was freely mobile while the reminder could have a role in maintaining the integrity of the TM and sensory structures as discussed above.

Assuming that some form of Ca^2+^equilibrium exists between the Ca^2+^ ducts and the stereocilia thanks to their attachments, the faster flow of stereocilia Ca^2+^ into the cell would drive the ducts’ Ca^2+^ into the stereocilia due to the Le Chatelier principle.

Together, the data described above indicate that the Ca^2+^ducts attach the TM to the stereocilia of IHCs and OHCs and support that the Ca^2+^ducts ensure these stereocilia access to TM Ca^2+^.

### Sound-evoked mechanical responses of the TM and stereocilia

The TM-embedment of the IHC stereocilia raises the question of how these stereocilia are stimulated.

To address this issue, we stained the TM (known to have a high negative charge-density^41^), with the cationic dye di-3-aneppdhq^10^ and then investigated the sound-evoked motions of same-location TM and stereocilia by high-speed confocal imaging^42^.

**Fig. 4:**
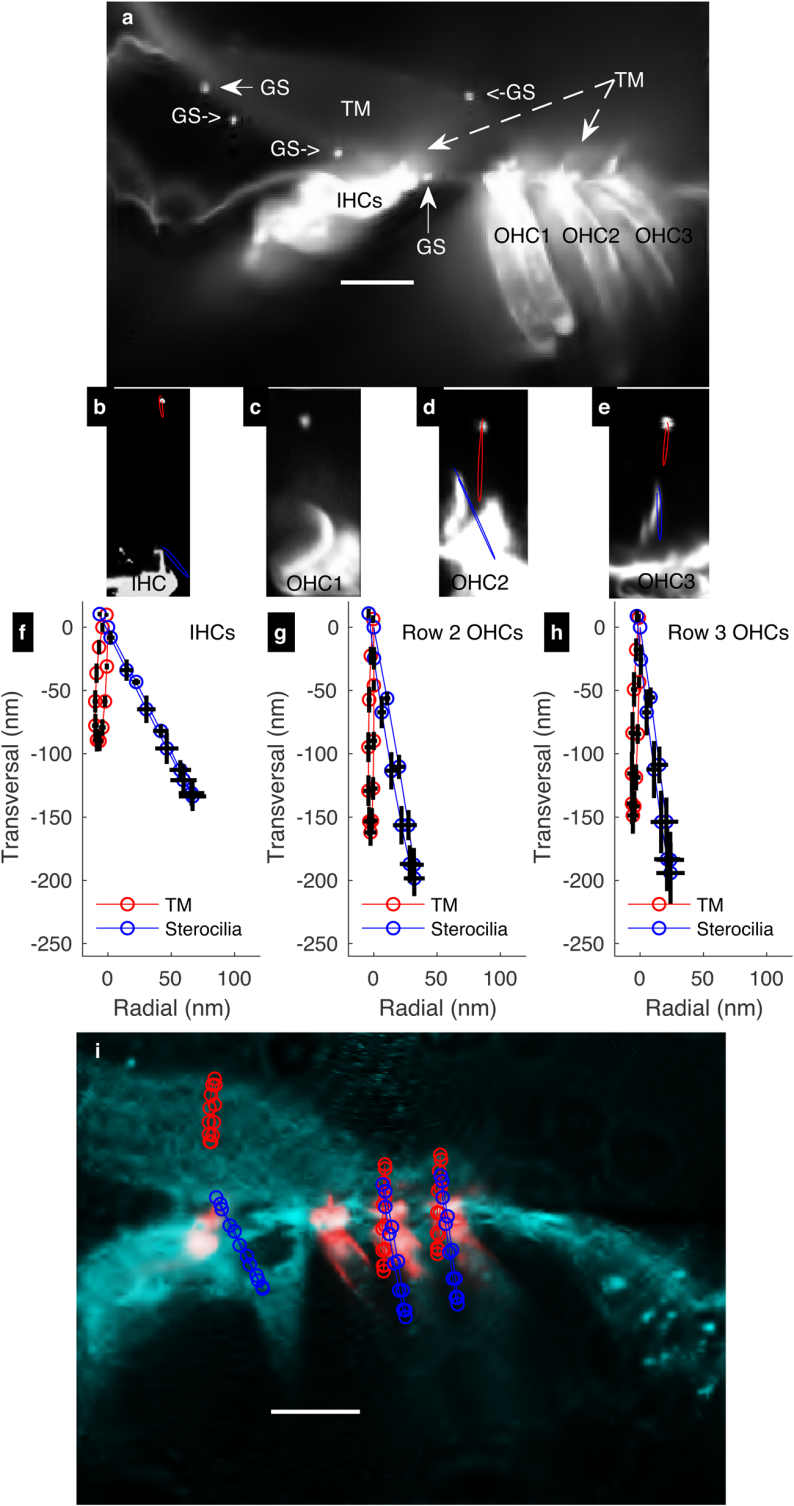
Sound-evoked movements of same-location TM and stereocilia bundles from IHCS and OHCS. **a** A confocal image of di-3-aneppdhq-stained OC revealed bright granular structures (GSs) scattered around the TM. These structures were used to track the sound-evoked movements of the TM. **b**–**e**. Confocal images of same-location TM and stereocilia bundles in IHCs, row 1 OHCs, row 2 OHCs, and row 3 OHCs, respectively. Example of individual sound-evoked motion trajectories associated with these structures at the best frequency (**b**, **d**, and **e**) are shown in red for the TM and blue for the stereocilia. **f**–**h** Sound-evoked motion trajectories at the best frequency for the TM (red) and same-location stereocilia bundle tip (bleu) in IHCs, row 2 OHCs, and row 3 OHCs, respectively as averaged in several preparations (solid line, mean; error bars, s.e.m.). For IHCs, *n* = 21 same-location TM and IHCs from nine preparations; for row 2 OHCs, *n* = 23 same-location TM and row 2 OHCs from 12 preparations; for row 3 OHCs *n* = 17 same-location TM and row 3 OHCs from 13 preparations. **i** The mean values for trajectory data from **f**–**h** (for *n* values, see the previous sentence) were replotted on the composite image from Fig. 2c (representative of ten different preparations) to highlight the OC regions where the motion measurements were performed. All scalebars 25 µm.

Figure 4a shows the fluorescence pattern across the di-3-aneppdhq-stained OC.

Di-3-aneppdhq-stained the hair cells and their stereocilia (Fig. 4a) whose morphology was similar to what is seen in vivo^29^.

Surprisingly, di-3-aneppdhq-stained also the TM (Fig. 4a), indicating that adjusting the microscope sensitivity can reveal more details in the TM (compare Figs. 4a and 2b). Although the TM staining was relatively weak, it was enough to see that the IHC and OHC stereocilia bundles were TM-embedded (Fig. 4a).

A close examination of the TM revealed 1 µm-wide granular structures (GSs) randomly distributed across the TM (Fig. 4a). Remarkably, one GS can be seen near the RL, supporting the TM-RL attachment (Fig. 4a).

We then used GS-stereocilia bundle pairs (Fig. 4b–e) to investigate the sound-evoked mechanical relationship between the TM and stereocilia in IHC and OHC regions by high-speed confocal imaging^42^, which allows for acquisition of 12 images at 12 equidistant phases of the acoustic stimulus. Row 1 OHC stereocilia bundles (Fig. 4c) were not included because their stereocilia bundles do not often appear perfectly straight when viewed in the cross-section orientation.

To quantify the sound-evoked motion of the TM and stereocilia, the image series above were analysed by optical flow algorithms in MATLAB^42^, which tracks and stores the coordinates of these structures across the acoustic phases.

Plotting these phase-dependent positions in a two-dimensional-space (transversal vs. radial directions) yield elliptical trajectories, for the TM (red trajectories, Fig. 4b, d, e) and stereocilia (blue trajectories, Fig. 4b, d, e) in the IHC and OHC regions. Trajectories from different preparations were then fitted with ellipse equations (see Methods) for statistical analysis of the associated parameters.

Figure 4f–h, show the averaged sound-evoked motion trajectories of the TM and stereocilia from the IHCs, row 2 OHCs, and row 3 OHCs, respectively when the intensity and the frequency of the sound stimulus were respectively ≈80 dB SPL and ≈200 Hz (the best frequency, BF). It was evident that the motion amplitude of the TM was smaller relative to same-location stereocilia. However, this behavior was a common feature in both the IHCs and OHCs as detailed below.

In the IHC region, the TM moved nearly transversally towards the BM, with a major peak-to-peak amplitude of 116 ± 7 nm (mean ± s.e.m., *n* = 21 same-location TM and IHCs from nine preparations, red plots, Fig. 4f). In the row 2 OHC region (Fig. 4g), the TM moved with an amplitude of 178 ± 11 nm (mean ± s.e.m.; *n* = 23 same-location TM and row 2 OHCs from 12 preparations) while in row 3 OHC region (Fig. 4h) the TM motion had a peak-to-peak amplitude of 183 ± 13 nm (mean ± s.e.m.; *n* = 17 same-location TM and row 3 OHCs from 13 preparations).

In agreement with past studies^24,25,43^, the motion trajectory at the stereocilia bundle tip was radially inclined and the major axis peak-to-peak amplitude was larger relative to the TM across the OC (Fig. 4f–h). In the IHC region (Fig. 4f, blue trajectory), the peak-to-peak motion amplitude of the stereocilia bundle tip was 180 ± 13 nm (mean ± s.e.m.; *n* = 21 same-location TM and IHCs from nine preparations) and the difference relative to the same-location TM counterpart was significant (*p* = 0.00006, Wilcoxon signed-rank test). In row 2 OHCs (Fig. 4g, blue trajectory), the stereocilia tip motion amplitude was 224 ± 14 nm (mean ± s.e.m.; *n* = 23, same-location TM and row 2 OHCs from 12 preparations), which was significantly larger relative to same-location TM counterpart (*p* = 0.00003, Wilcoxon signed-rank test). In row 3 OHC stereocilia (Fig. 4h, blue trajectory), the peak-to-peak motion amplitude of the stereocilia tip was 251 ± 22 nm (mean ± s.e.m.; *n* = 17 same-location TM and row 3 OHCs from 13 preparations), which in this case was also significantly larger compared to same-location TM motion amplitude (*p* = 0.0003, Wilcoxon signed-rank test).

Plotting the average trajectories on their approximative location on the composite image of the OC from Fig. 2c, confirms that the peak-to-peak motion amplitude for the TM was smaller relative to same-location bundle tip, irrespective of the hair cell type (Fig. 4i). Since our data above show that the TM and stereocilia are attached, the difference in their motion amplitudes indicates that the TM-stereocilia links stretched during sound stimulation.

**Fig. 5:**
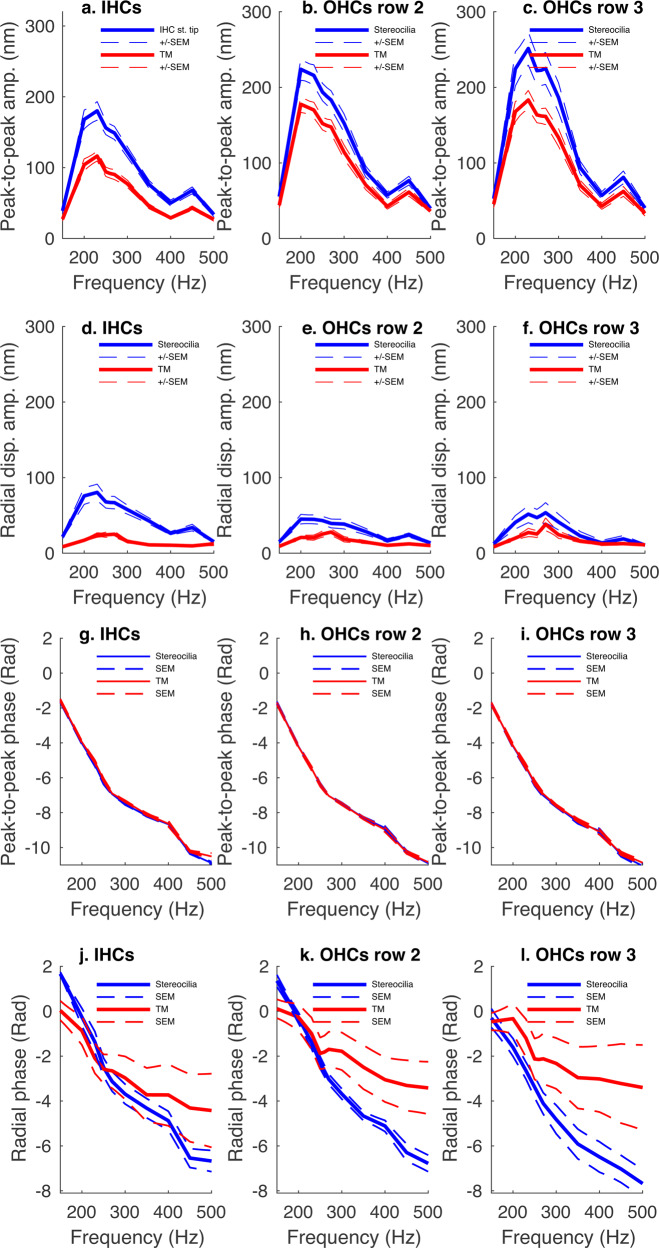
Frequency dependency of sound-evoked motion amplitude and phase of the TM and same-location IHC and OHC stereocilia bundles. **a**–**c** Peak-to-peak motion amplitude for the TM (red) and same-region stereocilia (blue) computed from the associated trajectories (see Fig. 4f–h) and across frequencies (solid lines, mean; error bars, s.e.m.). Source data are provided as a Source data file. **d**–**f** Radial motion amplitude vs. frequency as computed from the trajectories described above (solid lines, mean; error bars, s.e.m.). Source data are provided as a Source data file. **g**–**i** Frequency dependency motion phases of the TM (red) and same-location stereocilia (blue) as computed along the major axis of their respective trajectories described above (solid lines, mean; error bars, s.e.m). Source data are provided as a Source data file. **j**–**l** Same as above except that the phase plotted was the one associated with the radial component of the motion (solid lines, mean; error bar, s.e.m.). Detailed information about the *n* values is given in the main text. Source data are provided as a Source data file.

As shown by Fig. 5a–c, the stereocilia and TM responded to frequencies between 150 and 500 Hz and the motions were tuned. In addition, same-location TM and stereocilia had a similar BF, irrespective of the hair cell type. Although the BF exhibited small variations with values of 230–240 Hz for the IHC and row 3 OHC regions, and 200 Hz for the row 2 OHC region, this was clearly not a parameter that distinguished IHC from OHC regions (Fig. 5a–c). The differences between the TM and stereocilia motion amplitudes seen across frequency indicate that the TM-stereocilia links stretched in a frequency-dependent fashion both in the IHC and OHC regions.

Radially, the stereocilia motion amplitude remained larger relative to same-location TM (Fig. 5d–f). In the IHC region, the stereocilia motion amplitude was 80 ± 11 nm compared to 25 ± 4 nm for same-location TM (Fig. 5d, blue vs. red traces; mean ± s.e.m.; *n* = 21 same-location TM and IHCs from nine preparations; *p* = 0.0005, Wilcoxon signed-rank test). In the row 2 OHC region, the stereocilia motion amplitude was 45 ± 7 nm compared to 28 ± 4 nm for same-region TM (Fig. 5e, blue vs. red traces; mean ± s.e.m.; *n* = 23 same-location TM and row 2 OHCs from 12 preparations; *p* = 0.009, Wilcoxon signed-rank test). In the row 3 OHC region, the stereocilia motion amplitude was 54 ± 13 nm compared to 38 ± 9 nm for same-region TM (Fig. 5f, blue vs. red traces; mean ± s.e.m.; *n* = 17 same-location TM and row 3 OHCs from 13 preparations; *p* = 0.36, Wilcoxon signed-rank test). The larger IHC radial response is consistent with the fact that the cochlear amplifier boosts the IHC motion radially^43^.

Remarkably, the stereocilia radial motion BF compared poorly to that of the TM (Fig. 5d–f). In IHCs, the BF was 230 Hz for the stereocilia, compared to 270 Hz for same-location TM (Fig. 5d). In the row 2 OHC region, the stereocilia BF was 200 Hz compared to 270 Hz for same-location TM (Fig. 5e). In the row 3 OHC region, however, both the stereocilia and same-location TM had an identical BF of 270 Hz (Fig. 5f), and the higher BF could be explained by the larger distance from the pivot point. However, the radial motion BF was clearly not a good discriminator that distinguished the IHCs from the OHCs.

A key argument frequently advanced by supporters of the IHC stimulation by viscous drag is that the IHC vs. OHC motions exhibit a phase difference as large as 180°^15,44^, which would cause the IHCs and OHCs as well as the associated TM regions to move antiphasically^44–46^.

To address this issue, we examined the motion phases for the stereocilia bundle tip and same-location TM for frequencies between 150 and 500 Hz (Fig. 5g–i). The phase data were then analyzed with linear mixed modeling (LMM, see Methods).

For the IHC stereocilia bundle tip, the phase decreased from −1.61 ± 0.09 to −10.89 ± 0.08 rad (mean ± s.e.m.; *n* = 21 same-location TM and IHCs from nine preparations; Fig. 5g, blue trace). For same-location TM, the phase decreased from −1.56 ± 0.08 to −10.51 ± 0.19 rad (mean ± s.e.m.; *n* = 21 same-location TM and IHCs from nine preparations; Fig. 5g, red trace). The two traces were similar (*p* = 0.1, LMM, *n* = 21 same-location TM and IHCs from nine preparations). The phase-frequency function had a slope of −0.024 rad/Hz and the frequency dependency of the phase was statistically significant (*p* = 6 × 10^−211^, LMM, *n* = 21 same-location TM and IHCs from nine preparations).

In the row 2 OHC region, the stereocilia bundle and TM phases decreased from −1.71 ± 0.08 to −10.91 ± 0.11 rad (mean ± s.e.m.; *n* = 23 same-location TM and row 2 OHCs from 12 preparations; Fig. 5h, blue trace) and from −1.75 ± 0.07 to −10.83 ± 0.11 rad (mean ± s.e.m.; *n* = 23 same-location TM and row 2 OHCs from 12 preparations; Fig. 5h, red trace), respectively. The two phase-frequency functions overlapped (*p* = 0.8, LMM, *n* = 23 same-location TM and row 2 OHCs from 12 preparations). The associated slope was −0.024 rad/Hz and the frequency dependency of the phase was statistically significant (*p* = 1 × 10^−232^, LMM, *n* = 23 same-location TM and row 2 OHCs from 12 preparations).

In the row 3 OHC region, the phase for the stereocilia bundle and TM decreased from −1.76 ± 0.09 to −11.06 ± 0.15 rad (mean ± s.e.m. *n* = 17 same-location TM and row 3 OHCs from 13 preparations; Fig. 5i, blue trace) and from −1.77 ± 0.09 to −10.87 ± 0.14 rad (mean ± s.e.m. *n* = 17 same-location TM and row 3 OHCs from 13 preparations; Fig. 5i, red trace), respectively. The two phase-frequency functions were undistinguishable (*p* = 0.5, LMM, *n* = 17 same-location TM and row 3 OHCs from 13 preparations) and the associated slope was −0.024 rad/Hz. The frequency dependency of the phase was statistically significant (*p* = 2 × 10^−177^, LMM, *n* = 17 same-location TM and row 3 OHCs from 13 preparations).

These results show that during acoustic stimulation, IHC and OHC stereocilia experience a similar mechanical relationship with TM and are consistent with the stereocilia-TM-embedment in both the IHC and OHC regions.

Mechanical measurement on isolated TM fragments from the mice have suggested that the TM has its own longitudinally propagating traveling waves^47,48^ that could stimulate stereocilia radially to activate the MET channels. However, this has yet to be demonstrated in a functional cochlea.

To address this issue, we evaluated the radial motion phases of the stereocilia bundle and same-location TM for frequencies between 150 and 500 Hz (Fig. 5j–l). The data were then analyzed by LMM.

In the IHC region, the stereocilia phase decreased from 1.67 ± 0.08 rad to −6.67 ± 0.47 rad with a slope of −0.0231 rad/Hz (mean ± s.e.m.; *n* = 21 same-location TM and IHCs from nine preparations; Fig. 5j, blue trace). For same-location TM, the phase decreased from 0.034 ± 0.43 to −4.42 ± 1.64 rad with a slope of 0.0124 rad/Hz (mean ± s.e.m.; *n* = 21 same-location TM and IHCs from nine preparations; Fig. 5j, red trace). The slope difference between the two phase-frequency functions was statistically significant (*p* = 0.0001, LMM, *n* = 21 same-location TM and IHCs from nine preparations). In addition, the phase dependency in frequency and structure type were statistically significant with *p* = 1 × 10^−28^ and 0.001, respectively (LMM).

In the row 2 OHC region, the phase associated to the stereocilia decreased with a slope of −0.0228 rad/Hz from 1.36 ± 0.27 to −6.78 ± 0.37 rad (mean ± s.e.m.; *n* = 23 same-location TM and row 2 OHCs from 12 preparations; Fig. 5k, blue trace). For the same-location TM, the phase decreased with a slope of −0.0108 rad/Hz from 0.11 ± 0.42 to −3.42 ± 1.12 rad (mean ± s.e.m.; *n* = 23 same-location TM and row 2 OHCs from 12 preparations; Fig. 5k, red trace). The slopes associated with the two phase-frequency functions were statistically different (*p* = 2 × 10^−7^, LMM, *n* = 23 same-location TM and row 2 OHCs from 12 preparations). In addition, the effect of the frequency on the phase was statistically significant (*p* = 2 × 10^−36^, LMM).

In the row 3 OHC region, the phase associated to the stereocilia bundle decreased with a slope of −0.0212 rad/Hz from −0.30 ± 0.42 to −7.68 ± 0.63 (mean ± s.e.m.; *n* = 17 same-location TM and row 3 OHCs from 13 preparations; Fig. 5l, blue trace). For the TM, the phase decreased with a slope −0.0095 rad/Hz from −0.46 ± 0.38 to −3.40 ± 1.90 rad (mean ± s.e.m.; *n* = 17 same-location TM and row 3 OHCs from 13 preparations; Fig. 5l, red trace). These slopes were statistically different (*p* = 0.0002, LMM, *n* = 17 same-location TM and row 3 OHCs from 13 preparations). In addition, the effect of the frequency and structure type on the phase were statistically significant (*p* = 5 × 10^−19^, LMM).

Although various types of traveling waves have been shown to exist in the cochlea^49–52^, the radial phase data above constitute the first in situ demonstration for the existence of the TM own traveling waves^47^.

### How deep are the IHC stereocilia-TM-embedded?

To determine how deep the TM-stereocilia embedment was, we stained the OC with dextran-conjugated zFluor. According to the supplier (AAT Bioquest, USA), zFluor is much more photostable i.e., brighter than Cy5 for identical spectral characteristics.

**Fig. 6:**
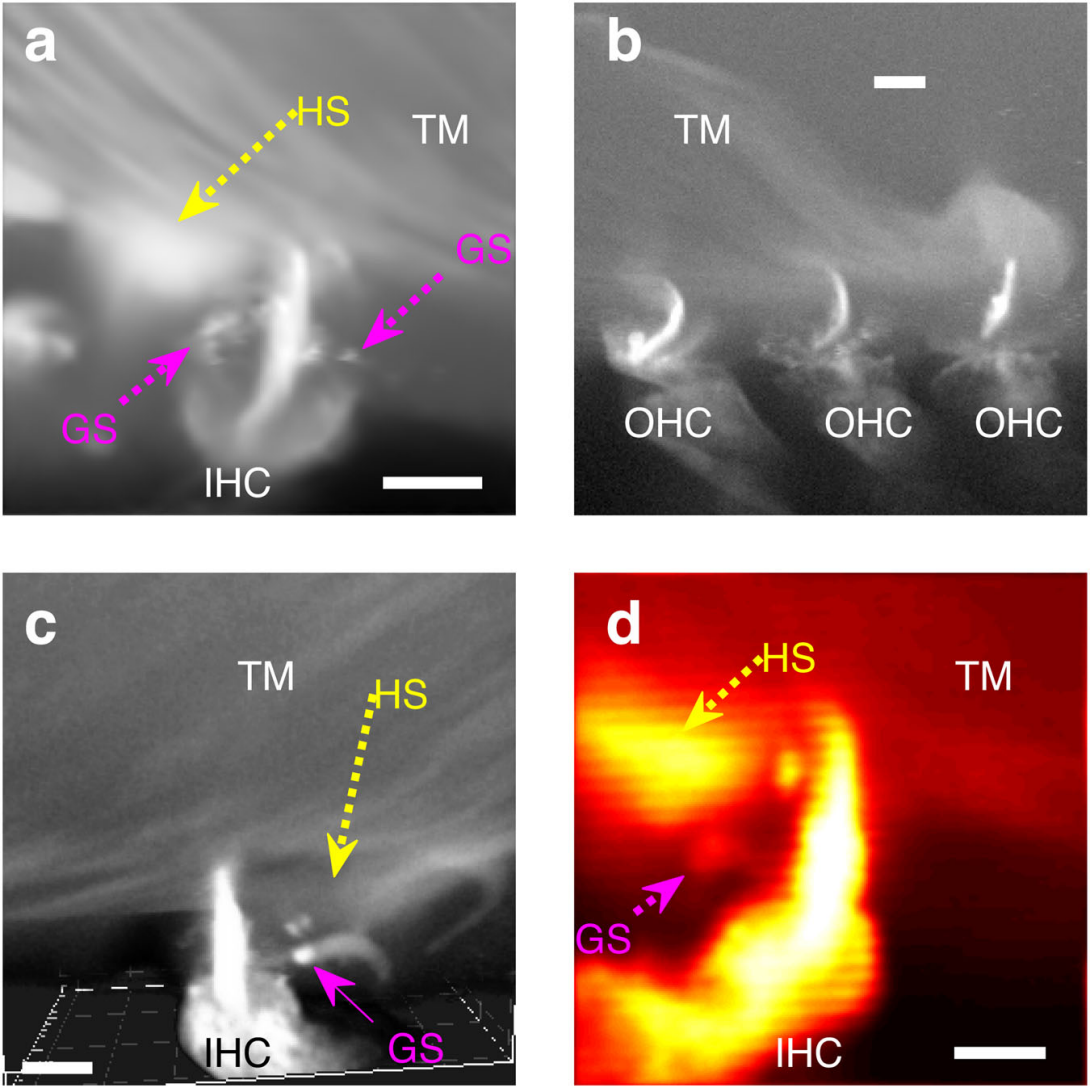
IHC stereocilia are fully TM-embedded. **a**, **b** The confocal images of the (dextran-conjugated) zFluor-stained OC show that zFluor stained the IHCs and OHCs as well as the TM. In addition, the zFluor fluorescence patterns revealed filamentous structures that contacted the stereocilia in similar fashion as the Ca^2+^ ducts, suggesting that the Ca^2+^ ducts had electrical properties that activated zFluor. The dye stained also the granular structures (GSs) as well as the Hensen’s stripe (HS) most likely due to the associated electrical environments. **c** 3D reconstruction of the IHC and the associated TM region. The 3D structure was rotated to show the opposite side of the view seen in **a**. Again, the GSs and Ca^2+^ duct-like structures can easily be visualized. In addition, some GSs surprisingly localized close to the cell’s cuticular plate, which is part of the RL, as seen in Fig. 4a, supporting that the TM-embedded the entire length of the IHC stereocilia bundles. The 3D reconstruction was prepared in Imaris 9.2 from 35 z-stacks with a z-step of 1 µm. **d** High-speed confocal image of the IHC stereocilia and the associated TM region captured during acoustic stimulation with a 1 kHz-tone, i.e., 5x their best frequency, and at 103 dB SPL. Again, the IHC stereocilia bundle can be seen TM-embedded even when stimulated with such a high frequency tone. The GS captured between the HS and the stereocilia bundle suggests that some GSs could have a connective role that links the HS to the base and tip of the stereocilia bundle. Data in **a**–**d** were representative of five different preparations. All scalebars 5 µm.

To our surprise, zFluor produced not only a strong labeling in the TM but also in the hair cell membrane as well as their stereocilia and cuticular plates (Fig. 6a–d).

Historically, only potentiometric dyes have been known to stain hair cells^53–55^. That zFluor produced a strong labeling both in the hair cell membranes, their cuticular plates and stereocilia suggests that zFluor could be a reliable potentiometric dye.

A close examination of the TM region revealed that zFluor stained the Hensen’s stripe as well as GSs similar to the ones seen in the confocal images of the di-3-aneppdhq-stained TM (Fig. 4). Remarkably, some of these GSs localized as low as the level of IHC cuticular plates, as seen in di-3-aneppdhq-stained TM images (compare Figs. 4a and 6), indicating that the full length of the IHC and OHC stereocilia bundles were embedded within the TM. Furthermore, zFluor stained TM structures that greatly resembled the Ca^2+^ ducts and which contacted the IHC and OHC stereocilia in a similar fashion as Ca^2+^ ducts (Fig. 6).

If zFluor stained the hair cells due to their membrane electrical properties as argued above, the staining of the GSs and Ca^2+^ ducts could reflect the existence of a unique electrical micro-environment in these structures, presumably conferred by Ca^2+^.

Overall, these zFluor imaging data are consistent with the data above that showed that the TM remained attached to the IHC stereocilia even when acoustically stimulated at 150–500 Hz.

To determine whether the TM-stereocilia links remained stable at even higher frequencies, we stimulated IHC stereocilia and same-location TM at 1 kHz and visualized the motion by high-speed confocal imaging^42^. This is 5x the BF for the cochlear region of interest (Fig. 5a–f). To ensure that these low frequency hearing structures responded with a visible motion to such a high frequency, the SPL was increased to 103 dB SPL.

As shown by the Supplementary Movie 3 and Fig. 6d, which is a still image extracted from this movie, the TM and the IHC stereocilia moved with different motion patterns while the stereocilia bundle remained fully embedded in the TM, which is consistent with the data described above.

To investigate whether the GSs were regularly distributed along the cochlear spiral, we systematically acquired confocal images of the zFluor-stained TM and IHC stereocilia (see Fig. 6a, c, d for the exact frame size) every single µm and up to 14–35 µm in five animals, giving us access to a total of 130 optical sections for the TM-IHC regions for GS quantification. Of these sections, 116 (or about 90% of them) had at least one GS and only 14 of them lacked a GS. In average, the number of GSs was 2.1 ± 0.1 per optical section (mean ± s.e.m.). These data suggest that the GSs were somewhat regularly distributed longitudinally.

We note that GS-like structures have been observed by immunogold electron microscopy in the TM from the guinea pig, with irregular size and position distribution, while some of them formed clusters that could be consistent with our GSs^56^.

## Discussion

In this study we have shown that Ca^2+^ ducts connect the TM to the stereocilia of IHCs and OHCs and that the TM-RL interface was tighter than previously thought. Specifically, the stereocilia of IHCs and OHCs were similarly TM-embedded. This similarity in stereocilia-TM relationship across hair cell types caused the IHC and OHC stereocilia to be phase-locked during sound stimulation. The IHC stereocilia remained TM-embedded even when the stimulus frequency was five times the BF, suggesting that the IHC stereocilia-TM links are stable at high frequencies.

Phylogenetically speaking, the emergence of TM elements some 320 million years ago has generally improved the hair cell frequency sensitivity compared to TM-less OCs^57^.

However, the TM tends to be more useful for high frequency than for low frequency hearing^57^, raising the question of why amniotes conserved the TM in their low frequency hearing regions if it was barely relevant?

The presence of Ca^2+^ ducts and the TM-stereocilia embedment enabling the TM to convey Ca^2+^ and mechanical stimuli directly to the hair cells could make the TM relevant for both low and high frequency hearing regions alike.

The presence of Ca^2+^ ducts could also address a prominent dichotomy that has long dodged the stereocilia: the resting MET channel P_open_ requires 40 to 150 µM in [Ca^2+^]^11^, which is substantially higher than available in endolymph (typically 20 µM)^10,58^. Ca^2+^ ducts could resolve this issue by directly funnelling high TM Ca^2+^ concentration to the stereocilia.

Interestingly, Ca^2+^ removal weakened and disrupted the attachments of the TM to the RL and stereocilia. It is already known that proteins such as stereocilin attach the TM to the OHC stereocilia^12^. However, the expression of otoancorin near the IHC cuticular plate^59^ suggests that this could be one of the proteins that attach the IHC stereocilia to the TM. Our study suggests that the function of these proteins could be Ca^2+^-dependent and will most likely spur interest into the Ca^2+^ dependency of their function.

While the exact molecular nature of the Ca^2+^ ducts will certainly be addressed by future studies, we also expect that their discovery will inspire research into the physiological role of the TM, whose function has been until recently thought to be mostly mechanical.

## Methods

### The temporal bone preparation from the guinea pig

This study complied with all relevant ethical regulations for animal testing and research. All the animal procedures described in this study were approved by the Regional Ethics Committee in Linköping, Sweden (Permit number DNR 5111-2019).

Young adult guinea pigs of either sex (Dunkin Hartley, 200–400 g i.e., 2 to 5 weeks old) were anesthetized with a sodium pentobarbital intraperitoneal injection (0.8 mL, 50 mg/mL) and then decapitated.

Immediately, the temporal bone was carefully and rapidly excised and mounted in a custom-built chamber, after which the bulla was gently opened with a fine scalpel and scissors in order to gain access to the cochlea. Right after this step, the entire preparation was immersed in an oxygenated (95% O_2_ and 5% CO_2_) cell culture medium (minimum essential medium with Earle’s balanced salts, room temperature). Because of the special design of the custom experimental chamber, the cochlea and the middle ear were immersed in the medium while the outer ear was shielded to allow for natural sound stimulation with a computer-controlled speaker inserted in the ear canal. With a fine scalpel, two small openings were made at the apex and base of the cochlea^10^. A plastic tube was then fitted into the basal opening to allow perfusion of scala tympani with the medium above gently flowing (∼0.6 ml/h) from an external (4 ml) tank. The apical opening allowed the perfusion medium to flow out and was used for confocal imaging and electrophysiological recording of sound-evoked responses of OC. Because of the immersion of the middle ear, the effective sound stimulus intensity decreased by at least 20 dB SPL^23^. The values given in the present paper were therefore corrected accordingly.

### Electrophysiology recording

For the recording of CM responses (Ix1 amplifier (Dagan Instruments) of the OC, a thin capillary glass (World Precision Instruments) was pulled with a standard puller and filled with an endolymph-mimicking solution containing 1.3 mM NaCl, 31 mM KHCO_3_, 128.3 mM KCl, and 0.023 mM CaCl_2_ (pH 7.4, osmolality 300 mOs/kg) as well as a fluorescent dye or EGTA (100 µM) where indicated and bevelled at an angle of 20° to 3–7 MΩ. The electrode was then made to carefully penetrate the Reissner’s membrane with a stepping manipulator. Dye and pharmacological agents dissolved in the electrolyte as above were administrated either electrophoretically or by a gentle pressure injection, where indicated, with a Picospritzer. The CM data acquisition was achieved using custom LabView software.

### Confocal imaging

The OC was imaged in the preparation above with a laser scanning confocal microscope (LSM 780 (ZEN 2012 black edition software), Zeiss, Germany). The microscope and the preparation are housed in a light protected Faraday enclosure stabilized by a vibration-damping table. A 40x, 0.8 NA water immersion lens (Nikon NIR APO) was used for reflected light confocal imaging of the OC and Ca^2+^ fluorescence confocal imaging with the ratiometric indicator Cal-520L^®^/Cy5-Dextran Conjugate also known as RatioWorks^TM^ (Cal-520L: excitation, 498 nm; emission, 521 nm; Cy5: excitation 651 nm; emission, 670 nm) as well the dextran-conjugated zFluor, where indicated (AAT Bioquest, USA). For sound-evoked motion measurements by high-speed confocal imaging^42^, the 0.8-NA water immersed lens was used after staining the stereocilia along with the TM with the membrane dye di-3-aneppdhq^10^ (Thermo Fischer).

When imaged under the cross-section view, the hair cells appear as a single IHC and three OHCs. In terms of coordinate, this z-position is not far from the injection site because this is generally where the electrode can smoothly penetrate the Reissner’s membrane i.e., when it is perpendicular to the Reissner’s membrane and parallel and close to the stria vascularis. Because the preparation rests on a rotative mount, this orientation is easy to find when the preparation is tilted such that the apical cochlear opening is optimally oriented relative to the lens.

For Ca^2+^ ratiometric imaging, Cal-520L/Cy5 dextran conjugate (40 µM in artificial endolymph) was loaded into a thin glass microelectrode. The indicator solution was then released from the electrode by application of a small pressure on the back end of the electrode (<4 psi, electrode impedance 3 MΩ) upon which the indicator rapidly partitioned in the TM and other water-accessible spaces from the injection site.

For Ca^2+^ratio quantification for the stereocilia bundles, we used the MATLAB-built in “drawpolygon” function to delimit the stereocilia in the reference images with successive clicks of the mouse until the contour of such a region of interest (ROI) is fully outlined. Then from the corresponding ratiometric images, we used the coordinates of these ROIs to extract and quantify Ca^2+^ ratio values for the stereocilia of the different hair cell types.

### Frap experiments

For Cal-520L FRAP experiments, the data acquisition was performed with the lens above. After marking 1 micron-wide ROIs at the center of the stereocilia bundle and in the Ca^2+^ duct-rich region nearby, 50 pre-bleaching images were acquired in fast scanning mode with no interframe delay before the ROIs were photobleached with maximum laser power and then immediately 300 post-bleaching images were acquired in fast scanning mode with no interframe delay. The FRAP data were then processed in MATLAB and individual FRAP traces were fitted with a single-phase exponential model^60^ for statistical analysis of FRAP data in different preparations.

### Sound-evoked motion analysis and trajectory fitting

The procedure for obtaining the sound-evoked trajectories^10,42^ for different points of interest are given throughout the text. In order to reliably quantify the sound-evoked motion amplitudes, phases and other parameters of interest for the TM and stereocilia in different preparations, sound-evoked motion trajectories from each individual measurement were fitted with the ellipse equation^61^ in MATLAB (The MathWorks) and were also subjected to sine wave fitting with software packages already used in other publications^10,60^.

### Quantification and statistical analysis

As the confocal microscope setup used in this study was optimized to work with the left ears, only one temporal bone preparation could be used per animal. Consequently, the number of animals and the number of preparations are the same and are understood to be interchangeable throughout the manuscript. The number of individual measurements as well as the number of preparations (= number of animals) is given throughout the text. For the frequency-tuning of sound-evoked motion amplitudes and associated phases, the motion measurements were performed for the 150–500 Hz. To optimize the frequency resolution especially near the BF where the motion is largest, the frequency steps were reduced to 20 Hz closer to the BF and maintained at 50 Hz outside this region. For figure plotting, the data were then subjected to the interp1 MATLAB function for interpolation every 10 Hz. Data processing, analysis, and quantification were performed using custom scripts in MATLAB (The MathWorks), unless indicated otherwise.

Different types of statistic tests were used throughout this study depending on the data sets considered.

The statistical analysis of the phase across frequency for the motion of the stereocilia and same-location TM structure types was performed on the data before the interpolation step above. Accordingly, it was necessary to take into account the fact that such measurements were performed repeatedly for these structures for several frequencies. Inevitably, such repetitions introduce correlations that have to be dealt with by LMM^62^. The random effect in the model was the experiment ID whereas the fixed effects were the frequency and structure type. The dependent variable was the phase. The same model was used for the different hair cell regions. Calculations were performed using the lme4 and nlme packages in RStudio (version 1.2.1335)^63^.

For statistical analysis of motion amplitudes of the stereocilia and same-location TM at a single frequency (i.e., the BF) and FRAP parameters for the IHC stereocilia and same-location TM, the kind of correlation issue described above doesn’t arise here. Consequently, for such situations, Wilcoxon signed-rank test was deemed appropriate for paired samples^25^, where indicated. For statistical analysis of Ca^2+^ ratios in the four different stereocilia types, Kruskal–Wallis test was used. These calculations were performed in MATLAB (The MathWorks).

### Reporting Summary

Further information on research design is available in the Nature Research Reporting Summary linked to this article.

## Supplementary information

Reporting Summary

Description of Additional Supplementary Files

Supplementary Movie 1

Supplementary Movie 2

Supplementary Movie 3

## Data Availability

All relevant data that support the findings of this study are included within this published article and/or its Supplementary Movies and the Source Data File. Any remaining data supporting the findings from this study are available from the corresponding author upon reasonable request. Source data are provided with this paper.

## Source data

Source Data
