# Supplementary material for: Inner hair cell stereocilia are embedded in the tectorial membrane
Source: Nat Commun. 2021 May 10;12:2604. doi: 10.1038/s41467-021-22870-1 (PMC8110531; doi:10.1038/s41467-021-22870-1)
Supplement: Supplementary file 1 — Reporting Summary [file 41467_2021_22870_MOESM1_ESM.pdf]

nature research

Corresponding author(s): Pierre Hakizimana

Last updated by author(s): Mar 18, 2021

Reporting Summary

Nature Research wishes to improve the reproducibility of the work that we publish. This form provides structure for consistency and transparency in reporting. For further information on Nature Research policies, see our Editorial Policies and the Editorial Policy Checklist.

Statistics

For all statistical analyses, confirm that the following items are present in the figure legend, table legend, main text, or Methods section.

n/a	Confirmed	
☐	☒	The exact sample size (*n*) for each experimental group/condition, given as a discrete number and unit of measurement
☐	☒	A statement on whether measurements were taken from distinct samples or whether the same sample was measured repeatedly
☐	☒	The statistical test(s) used AND whether they are one- or two-sided<br>*Only common tests should be described solely by name; describe more complex techniques in the Methods section.*
☐	☒	A description of all covariates tested
☐	☒	A description of any assumptions or corrections, such as tests of normality and adjustment for multiple comparisons
☐	☒	A full description of the statistical parameters including central tendency (e.g. means) or other basic estimates (e.g. regression coefficient) AND variation (e.g. standard deviation) or associated estimates of uncertainty (e.g. confidence intervals)
☐	☒	For null hypothesis testing, the test statistic (e.g. *F*, *t*, *r*) with confidence intervals, effect sizes, degrees of freedom and *P* value noted<br>*Give P values as exact values whenever suitable.*
☒	☐	For Bayesian analysis, information on the choice of priors and Markov chain Monte Carlo settings
☒	☐	For hierarchical and complex designs, identification of the appropriate level for tests and full reporting of outcomes
☒	☐	Estimates of effect sizes (e.g. Cohen's *d*, Pearson's *r*), indicating how they were calculated

Our web collection on statistics for biologists contains articles on many of the points above.

Software and code

Policy information about availability of computer code

Data collection	LabView 2014 and ZEN 2012 black edition software
Data analysis	Matlab R2018b and RStudio (version 1.2.1335)

For manuscripts utilizing custom algorithms or software that are central to the research but not yet described in published literature, software must be made available to editors and reviewers. We strongly encourage code deposition in a community repository (e.g. GitHub). See the Nature Research guidelines for submitting code & software for further information.

Data

Policy information about availability of data

All manuscripts must include a data availability statement. This statement should provide the following information, where applicable:

- Accession codes, unique identifiers, or web links for publicly available datasets
- A list of figures that have associated raw data
- A description of any restrictions on data availability

All relevant data that support the findings of this study are included within this published article and/or its Supplementary Movies and the Source Data File. Any remaining relevant data supporting the findings of this study are available from the corresponding author upon reasonable request.

Field-specific reporting

Life sciences study design

All studies must disclose on these points even when the disclosure is negative.

Sample size	The parameters assessed in this study are frequently tested in the lab. Therefore, the choice of the sample size was guided by our longstanding experience with these experimental designs: Prasad et al, Commun Biol, 2020; DOI: 10.1038/s42003-020-01506-y; Strimbu et al, PNAS, 2019, DOI: 10.1073/pnas.1805223116 ; Hakizimana et al, Nat Commun, 2012; doi: 10.1038/ncomms2100
Data exclusions	Pre-established exclusion criteria was established: when the dissection accidentally goes wrong, the preparations get damaged and unresponsive and such preparations are not included
Replication	All the experiments were performed in several preparations and in each preparation, the measurements were performed on different hair cells or locations where indicated. Different types of statistic tests were used throughout this study depending on the data sets considered.<br><br>For the statistical analysis of the phase across frequency for the motion of the stereocilia and same-location TM structure types, it was necessary to take into account the fact that such measurements were performed repeatedly for these structures for several frequencies. Inevitably, such repetitions introduce correlations that have to be dealt with by linear mixed modelling. The random effect in the model was the experiment ID whereas the fixed effects were the frequency and structure type. The dependent variable was the phase. The same model was used for the different hair cell regions. Calculations were performed using the lme4 and nlme packages in RStudio (version 1.2.1335)68.<br><br>For statistical analysis of motion amplitudes of the stereocilia and same-location TM at a single frequency (i.e., the BF) and FRAP parameters for the IHC stereocilia and same-location TM, the kind of correlation issue described above doesn't arise here. Consequently, for such situations, Wilcoxon signed rank test was deemed appropriate for paired samples, where indicated. For statistical analysis of Ca2+ ratios in the four different stereocilia types, Kruskal-Wallis test was used. These calculations were performed in MATLAB (The MathWorks).<br>The differences were considered significant if $P<0.05$.
Randomization	Randomization was not relevant here because samples were not allocated into any study groups
Blinding	Not relevant because this was not a randomized study

Reporting for specific materials, systems and methods

We require information from authors about some types of materials, experimental systems and methods used in many studies. Here, indicate whether each material, system or method listed is relevant to your study. If you are not sure if a list item applies to your research, read the appropriate section before selecting a response.

Materials & experimental systems

n/a	Involved in the study
☒	☐ Antibodies
☒	☐ Eukaryotic cell lines
☒	☐ Palaeontology and archaeology
☐	☒ Animals and other organisms
☒	☐ Human research participants
☒	☐ Clinical data
☒	☐ Dual use research of concern

Methods

n/a	Involved in the study
☒	☐ ChIP-seq
☒	☐ Flow cytometry
☒	☐ MRI-based neuroimaging

Animals and other organisms

Policy information about studies involving animals; ARRIVE guidelines recommended for reporting animal research

Laboratory animals	Young adult guinea pigs of either sex (Dunkin Hartley, 2 to 5 weeks old (200 – 400 g))
Wild animals	The study did not involve wild animals.
Field-collected samples	The study did not involve samples collected from the field.
Ethics oversight	All the animal procedures were approved by the Regional Ethics committee in Linköping, Sweden (Permit number DNR 5111-2019)

Note that full information on the approval of the study protocol must also be provided in the manuscript.
