# Supplementary material for: Inner hair cell stereocilia are embedded in the tectorial membrane
Source: Nat Commun. 2021 May 10;12:2604. doi: 10.1038/s41467-021-22870-1 (PMC8110531; doi:10.1038/s41467-021-22870-1)
Supplement: Supplementary file 2 — Description of Additional Supplementary Files [file 41467_2021_22870_MOESM2_ESM.pdf]

Description of Additional Supplementary Files

Supplementary Movie 1. Probing the strength of the TM-RL attachments by reflected light confocal imaging. To determine whether the TM was loosely resting on the RL or whether the two structures attached firmly to each other, the OC was stimulated with loud sound (103 dB SPL near the best frequency). As the acoustic overstimulation caused the OC to contract towards the BM (@ the 3 s-time point in the movie), the TM was pulled along with the RL including in the inner and outer hair cell regions without revealing a gap along the TM-RL interface. A gap is only briefly seen when the acoustic overstimulation stopped (between the 6 and 7 s-time points in the movie) and recovered shortly afterwards. Confocal images were acquired continuously in the reflected light mode every second and the resulting image timeseries exported as a movie at 10 frames per second.

Supplementary Movie 2. The TM-RL attachments are Ca^{2+} -dependent. To determine the effect of Ca^{2+} removal on the integrity of the TM and its attachments to the RL, EGTA (100 µM) was briefly injected through a thin glass electrode inserted through an intact Reissner's membrane (see Methods) while the TM and the OC were monitored by reflected light confocal imaging. Before EGTA injection (between the 0 and 2 s-time points in the movie), the TM can be seen resting on the RL. Upon EGTA injection by application of a small pressure on the back end of the electrode (<4 psi, electrode impedance 3 Mega-ohms) at the 3 s-time point in the movie, the TM swelled rapidly and exposed a gap along its interface with the RL before this gap started to recover. A near full recovery can be seen between the 40 and 52 s-time points in the movie. Injection of more EGTA caused the TM to rapidly swell again (between the 53 and 57 s-time points in the movie), exposing again a large gap at its interface with the RL. Immediately, the OC was subjected to acoustic overstimulation (103 dB SPL near the best frequency). The contraction of the OC towards the BM caused the TM to detach free from the RL (see between the 57 and 58 s-time points in the movie). The interruption of the acoustic overstimulation allowed the TM-RL attachments to recover in the inner hair cell region (see at the 1 min 2 s-time point in the movie). Resuming the acoustic overstimulation did not cause this newly recovered TM-RL attachments to break (see between the 1 min 3 s and 1 min 6 s-time points in the movie), indicating that they had regained their normal strength. To determine whether the newly recovered TM-RL attachments were still Ca^{2+} -dependent, more EGTA was injected (at the 1 min 12 s-time point in the movie) and caused again the TM to swell rapidly (see between the 1 min 13 s and 1 min 17 s-time points in the movie). The contraction of the OC upon acoustic overstimulation (see at the 1 min 18 s-time point in the movie) caused the TM to break-free from the RL (see from the 1 min 18 s-time point in the movie) as the TM retreated towards the spiral limbus region. The confocal images were acquired continuously every second and the image time series was exported as a movie at 10 frames per second.

Supplementary Movie 3. **The IHC stereocilia remain TM-embedded at 5X the best frequency**. The (dextran-conjugated) zFluor-stained IHC stereocilia and the associated TM were stimulated with a 1 kHz-tone burst while a movie was acquired by a high-speed confocal imaging (Jacob et al., 2007). The original movie was looped several times to facilitate visualization. Because the IHC was from a cochlear region tuned to a best frequency of about 200 Hz, the stimulus with a tone burst of 1 kHz amounted to a higher frequency stimulation. Since this stereocilia bundle location does not normally respond to such a high frequency, the stimulus level had to be increased to 103 dB SPL in order obtain a visible motion. For comparison, if such a stimulus level was applied near the best frequency, the OC would have been severely damaged (see Suppl. Mov. 1).
